# Prevalence and risk factors of gestational diabetes in the health region of Lleida: a retrospective observational cohort study

**DOI:** 10.1007/s40618-023-02120-5

**Published:** 2023-06-18

**Authors:** M. Orós, D. Perejón, M. C. Serna, J. Siscart, J. Leon, M. Ortega, B. Salinas-Roca

**Affiliations:** 1 Institut d’Investigació en Atenció Primària IDIAP Jordi Gol, Institut Català de la Salut, Lleida, Spain; 2 Centre de Salut Eixample, Institut Català de la Salut, Lleida, Spain; 3 Departament de Medicina Familiar, Universitat de Lleida, Lleida, Spain; 4 Departament d’Endocrinologia i Nutrició, Hospital Universitari Arnau de Vilanova, Lleida, Spain; 5 Grup de Recerca Terapèutica en Atenció Primària (GRETAPS), Institut Català de la Salut, Lleida, Spain; 6 Grup d’investigació en Immunologia i Metabolisme (GRIM), Institut de Recerca Biomèdica, Lleida, Spain; 7 Department of Nursing and Physiotherapy, University of Lleida, Montserrat Roig 2, 25198 Lleida, Spain; 8 Global Research On Wellbeing (GRoW) Research Group, Blanquerna School of Health Science, Ramon Llull University, Padilla, 326-332, 08025 Barcelona, Spain

**Keywords:** Gestational diabetes mellitus, Immigrant women, Obesity, Population-based study

## Abstract

**Background:**

Diabetes is a very common metabolic condition during pregnancy. The number of cases increases with age and obesity. The prevalence of pre-gestational diabetes and gestational diabetes (GD) differs between different ethnic groups.

**Objective:**

The aim of the study was to analyse the prevalence of pre-gestational diabetes and GD in the health region of Lleida. We also studied the GD risk factors during pregnancy according to the country of origin of the pregnant woman.

**Methods:**

We performed a retrospective observational cohort study among pregnant women between 2012 and 2018 in the health region of Lleida. A multivariate model was performed with the different variables analysed by calculating the regression coefficient and its 95% confidence interval (CI).

**Results:**

In our sample of 17,177 pregnant women, we observed a prevalence of pre-gestational diabetes and GD of 8.2% and 6.5%, respectively. We found a relationship of gestational diabetes with different factors: age, with 6.8% in 30–34 year-old women and 11.3% in women over 35 (OR 1.78 and 3.29, respectively); overweight, with 8.29% (OR 1.89); and obesity, with 12.9% (OR 3.15). Finally, women from Asia and the Middle East and the Maghreb had a higher risk of diabetes, with 12.2% (OR 2.1) and 9.91% (OR 1.3), respectively, and Sub-Saharan women had a lower risk of it 6.07% (OR 0.71).

**Conclusions:**

GD has different risk factors, such as age, overweight, and obesity. Non-related conditions include hypothyroidism, arterial hypertension, and dyslipidaemia. Finally, pregnant women from the Maghreb, and Asia and the Middle East, are at higher risk of developing diabetes during pregnancy; meanwhile, Sub-Saharan origin is protector factor.

## Background

Hyperglycaemia during pregnancy includes pre-gestational (diagnosed before pregnancy), overt diabetes (hyperglycemia first recognised during pregnancy which meets the thresholds of diabetes in non-pregnant adults) and gestational (diagnosed during pregnancy) diabetes. The prevalence of the former among pregnant women is 1–12% [1], while gestational diabetes (GD) is the most common metabolic complication during pregnancy [2] and affects about one in six women (17%) [3]. It usually occurs in the middle of the pregnancy period, approximately between 24 and 28 weeks of gestation [4]. Both pre-gestational and gestational diabetes have been associated with the risk of maternal–foetal complications [5–12]. In particular, GD is associated with an increased risk of perinatal complications and the development of diabetes by the mother and her offspring [13, 14].

The aetiology of diabetes is multifactorial and not fully defined. However, in most cases, hyperglycaemia is the result of impaired glucose tolerance either due to dysfunction of pancreatic β cells, or to defects in the insulin signalling cascade that causes chronic insulin resistance [15, 16]. During pregnancy, high levels of diabetic hormones (prolactin, placental lactogen, progesterone, and cortisol) increase peripheral insulin resistance. In addition, there is an increase in energy demand and insulin to increase the body mass. As a result of insulin resistance, glucose intolerance appears, and the pancreas tries to fight insulin resistance by increasing its production. Finally, GD develops in women with a dysfunction in this cascade [17].

The incidence of type 1 and type 2 diabetes among women in reproductive age is growing globally, together with the incidence of obesity in the population and the average age of pregnant women. Diabetes has been linked to other prevalent conditions during pregnancy, although there are no conclusive studies to date. These conditions include hypothyroidism, which is the second most common medical condition during pregnancy, hypertension, and dyslipidaemia [18–20]. Finally, the country of origin of the pregnant woman is among the factors determining the prevalence of GD. Indeed, women from Maghreb [11, 12, 21] and Asia and the Middle East [22–29] are at higher risk of developing this metabolic disease. Spain has seen significant changes in the percentage of births, fertility, and immigration. The birth rate fell by 24.6% and the fertility rate (children per woman) decreased from 1.4 to 1.3 (1.2 for natives and 1.7 for immigrants) in 2018. The proportion of births among immigrant women has increased. In Catalonia, the percentage of live births was 70.4% from Spanish women and 29.6% from immigrant women [30].

There are few studies that analyse the prevalence of pre-gestational and gestational diabetes in our environment, as well as risk factors associated with the disease during pregnancy. Moreover, the increase in immigration in recent decades in Spain led to a concomitant increase in the number of pregnant women from different populations. In this context, our study had the aim of studying the prevalence of pre-gestational and gestational diabetes in the health region of Lleida and gestational diabetes risk factors, according to the different ethnic groups.

## Methods

### Study design and data collection

We conducted a retrospective cohort observational study among pregnant women between 2012 and 2018 in the health region of Lleida. We included patients who gave birth at the Arnau University Hospital in Vilanova de Lleida between 1 January 2012 and 31 December 2018. Data were obtained through the CMBD (“Conjunt Mínim de Base de dades”), the eCAP computerised medical history database, and the database of the Catalan Health Service. The latter collects the data of the prescriptions from the Social Security.

### Participants

We studied women who have given birth between 1 January 2012 and 31 December 2018. We collected pregnancy data from the date of the last period until the date of delivery. Women who did not belong to the health region of Lleida were excluded. To evaluate the representation of the sample, we calculated the percentage of pregnant women studied (registered at the Arnau University Hospital in Vilanova de Lleida) with respect to the total number of pregnant women in the Lleida health region (registered in the database of the “Institut estadístic de Catalunya”, Idescat) (Table 1).

**Table 1:** Number of pregnant women in the health region of Lleida by years, number of pregnant women in the sample, and percentage of the latter with respect to the former

Year	Part of the sample	Part of Idescat	Idescat/sample (%)
2012	3635	3788	90
2013	3370	3535	89
2014	3308	3592	86
2015	3162	3426	86
2016	3180	3283	90
2017	3034	3197	88
2018	3001	3029	93

The main variable was the presence of pre-gestational or gestational diabetes, and the diagnosis was done with the O'Sullivan test. This test consists in the determination of plasma glycaemia 1 h after the oral administration of 50 g of glucose. Diabetes was diagnosed when blood glucose was above 140 mg/dl (7.8 mmol/l). In this case, the diagnosis was confirmed by Oral Glucose Tolerance Test (OGTT) The OGTT was performed between 24 and 28th gestation weeks with 100 g of glucose and the subsequent determination of blood glucose at the beginning and after the 1st, 2nd, and 3rd hour [17]. The eCAP diabetes registration code corresponds to the CIE-10 code O24.9. Other variables, registered at the early pregnant period, were the age of the pregnant woman; her BMI; hypothyroidism (code E03.9 and E02 of the ICD-10); hypertension (code I10–I16 of the ICD-10); dyslipidaemia (code E78 of the ICD-10); depression (codes F32.0–F32.9, F33.0–F33.3, F33.8, F33.9, F34.1, and F41 0.2 of the ICD-10); and the region of origin (Latin America, Asia and the Middle East, Europe, Eastern Europe, and Maghreb) [31, 32].

### Statistical analyses

Numerical variables are described using the mean and the standard deviation, while categorical variables are described using absolute and relative frequencies. Differences between DM groups were assessed using an ANOVA or the Chi-square test, depending on whether the variables were quantitative or categorical, respectively. The risk of developing gestational diabetes according to the different variables was evaluated using a multivariate logistic regression using the diagnosis of DM during pregnancy as the response variables and the rest of the variables as predictors. Odds Ratios with the respective 95% confidence intervals were calculated. Statistical significance was established at *p* < 0.05. The analysis was carried out with R software, version 4.1.2.

### Ethical aspects

This study was approved by the ethics and clinical research committee “Institute for Primary Health Care Research Jordi Gol i Gurina (IDIAPJGol)” under the code 19/194-P. The study was conducted in accordance with the principles of the Declaration of Helsinki. We performed a pseudonymised retrospective descriptive cross-sectional study according to Additional Provision 17.2.d LOPD-GDD for research purposes, without the need to obtain the consent of the data holders. There was a technical and functional separation between the research team and the performer pseudonymisation, and data are only accessible to the research team. Technical measures have been taken to prevent re-identification and access by third parties through the CMBD database (“Conjunt Minim de Base de Dades”) the E-CAP computerised medical history database and the Catalan Health Service database.

## Results

### Epidemiological data

**Fig. 1:**
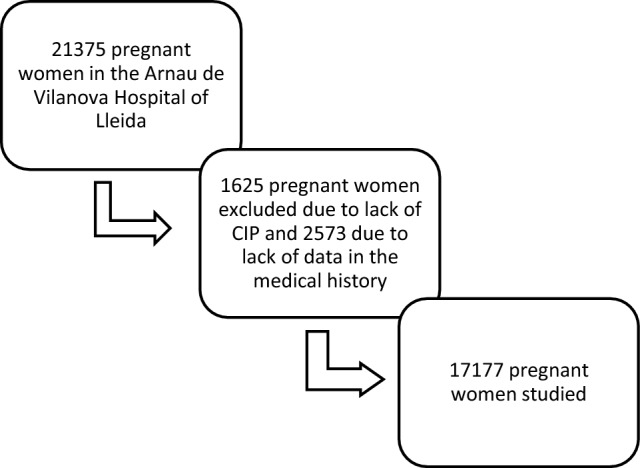
Sample of pregnant women studied

A sample of 21,375 pregnant women who had given birth at the Arnau University Hospital in Vilanova de Lleida between 2012 and 2018 (both inclusive) was obtained. We excluded 1625 women who did not have a personal identification code (CIP), as well as 2573 women that lacked multiple clinical history data. As a consequence, the final sample was of 17,177 patients (Fig. 1).

17,177 pregnant women were analysed, the distribution was 12–16% of the sample per year of study. The prevalence of pregnant women with diabetes (pre-gestational diabetes and GD) was 8.2% and GD 6.5%. A percentage of 40.6% of the mothers were < 30 years, and 21.3% equal to or older than 35 years. 24.9% were overweight (BMI 25–30) and 15.0% were obese (BMI ≥ 30). 6.6% of pregnant women had hypothyroidism, 2.3% arterial hypertension, 1.1% dyslipidaemia, and 2.5% depression. Moreover, pregnant women had different ethnic origins: 63.0% were from Western Europe, 14.9% from the Maghreb, 10.2% from Eastern Europe, 5.6% from Sub-Saharan Africa, 4.8% from Latin America, and 1.5% from Asia and the Middle East (Table 2).

**Table 2:** Characteristics of the studied population

	*N = *17,177
Year of delivery	17,177
2012	2740 (15.9%)
2013	2525 (14.7%)
2014	2491 (14.5%)
2015	2419 (14.1%)
2016	2418 (14.1%)
2017	2317 (13.5%)
2018	2224 (12.9%)
Age at pregnancy	17,177
< 30	6981 (40.6%)
30–35	6543 (38.1%)
> 35	3653 (21.3%)
BMI	16,803
≤ 25 kg/m^2^	10,112 (60.2%)
26–30 kg/m^2^	4177 (24.9%)
≥ 30 kg/m^2^	2514(15.0%)
Number of pregnancies	17,177
1	9009 (52.4%)
2	5181 (30.2%)
3	1870 (10.9%)
4	471 (2.74%)
> 4	471 (2.7%)
Preeclampsia	17,177
No	17,018 (99.1%)
Yes	159 (0.9%)
Multiple pregnancies	17,177
No	17,145 (99.8%)
Yes	32 (0.2%)
Caesarean section	17,177
No	14,201 (82.7%)
Yes	2976 (17.3%)
Duration of the pregnancy (qualitative)	12,962
Abortion	569 (4.4%)
Pre-term	769 (5.9%)
Full-term	11,296 (87.2%)
Prolonged	328 (2.5%)
Pregnancy risk	15,333
Risk free	7578 (49.4%)
Medium risk	4527 (29.5%)
High risk	2912 (19.0%)
Very high risk	316 (2.1%)
Pre-gestational diabetes mellitus tipus 1	17,177
No	17,124 (98.7%)
Yes	53(0.31%)
Pre-gestational diabetes mellitus tipus 2	17,177
No	16,044 (93.4%)
Yes	1123 (6.5%)
Overt diabetes mellitus	
No	17,166 (99.94%)
Yes	10 (0.06%)
Gestational diabetes	17,177
No	16,044 (93.4%)
Yes	1123 (6.5%)
Hypothyroidism	17,177
No	16,050 (93.4%)
Yes	1127 (6.6%)
Arterial hypertension	17,177
No	16,778 (97.7%)
Yes	399 (2.3%)
Dyslipidaemia	17,177
No	16,990 (98.9%)
Yes	187 (1.1%)
Depression	17,177
No	16,741 (97.5%)
Yes	436 (2.5%)
Region of origin	15,006
Sub-Saharan Africa	840 (5.6%)
Latin America	717 (4.8%)
Asia and the Middle East	222 (1.5%)
Western Europe	9461 (63.0%)
Eastern Europe	1533 (10.2%)
Maghreb	2233 (14.9%)
Newborn weight	15,133
No Macrosomia	14,113 (93.3%)
Macrosomia	1020 (6.7%)
APGAR test at the first minute	15,085
≥ 7	14,970 (97.5%)
< 7	379 (2.5%)
APGAR test at the fifth minute	15,087
≥ 7	14,970 (99.2%)
< 7	117 (0.8%)

### Risk factors for diabetes in pregnancy

Pre-gestational and gestational diabetes were more common among older women: 14.2% of pregnant women over the age of 35 had diabetes, in comparison to 4.4% under the age of 30. This difference was statistically significant. Another significantly different variable was the BMI: 16.1% of pregnant women with obesity presented diabetes, in comparison to 5.3% with normal weight. Moreover, 11.1% of patients with hypothyroidism had diabetes, in comparison to 7.9% of patients without hypothyroidism; 19.3% of hypertensive patients were also diabetics, in comparison to 7.9% of non-hypertensive patients; and 15.0% of patients with dyslipidemia had diabetes, in comparison to 8% of patients without dyslipidemia. In the analysis of the region of origin, statistically significant differences were also evident, with a higher proportion of gestational diabetes among women from Asia and the Middle East (12.2%), and from the Maghreb (9.9%) (Table 3).

**Table 3:** Relationship of diabetes with different variables

	No diabetes	Pre-gestational diabetes	Gestational diabetes	*p*
	*N = *15,773	*N = *271	*N = *1123	
Age at pregnancy				< 0.001
< 30	6675 (95.6%)	42 (0.6%)	262 (3.75%)	
30–34	5965 (91.2%)	124 (1.9%)	448 (6.85%)	
≥ 35	3133 (85.8%)	105 (2.88%)	413 (11.3%)	
BMI				< 0.001
≤ 25 kg/m2	9574 (94.7%)	103 (1.02%)	433 (4.28%)	
26–30 kg/m2	3748 (89.8%)	79 (1.89%)	346 (8.29%)	
> 30 kg/m^2^	2105 (83.9%)	80 (3.19%)	325 (12.9%)	
Number of pregnancies				< 0.001
1	8353 (92.8%)	75 (0.83%)	576 (6.40%)	
2	4743(91.6%)	114 (2.20%)	322 (6.22%)	
3	1682 (90.0%)	48 (2.57%)	138 (7.39%)	
4	573 (88.8%)	20 (3.10%)	52 (8.06%)	
> 4	422 (89.6%)	14 (2.97%)	35 (7.43%)	
Hypothyroidism				< 0.001
No	14,773 (92.1%)	241 (1.50%)	1028 (6.41%)	
Yes	1000 (88.9%)	30 (2.67%)	995 (8.44%)	
Arterial hypertension				< 0.001
No	15,452 (92.1%)	247(1.47%)	1070 (6.38%)	
Yes	321 (80.7%)	24(6.03%)	53 (13.3%)	
Dyslipidaemia				< 0.001
No	15,614 (92.0%)	262(1.54%)	1104 (6.50%)	
Yes	159 (85.0%)	9(4.81%)	19 (10.2%)	
Depression				1.000
No	15,373 (91.9%)	263(1.57%)	1095 (6.54%)	
Yes	400 (91.7%)	8(1.83%)	28 (6.42%)	
Region of origin				< 0.001
Sub-Saharan Africa	768 (91.4%)	21(2.50%)	51 (6.07%)	
Latin America	673 (93.9%)	3(0.42%)	41 (5.72%)	
Asia and the Middle East	190 (85.6%)	5(2.25%)	27 (12.2%)	
Europe	8683 (91.8%)	142(1.50%)	632 (6.68%)	
Eastern Europe	1431 (93.5%)	11(0.72%)	89 (5.81%)	
Maghreb	1971 (88.4%)	38(1.70%)	221 (9.91%)	

**Fig. 2:**
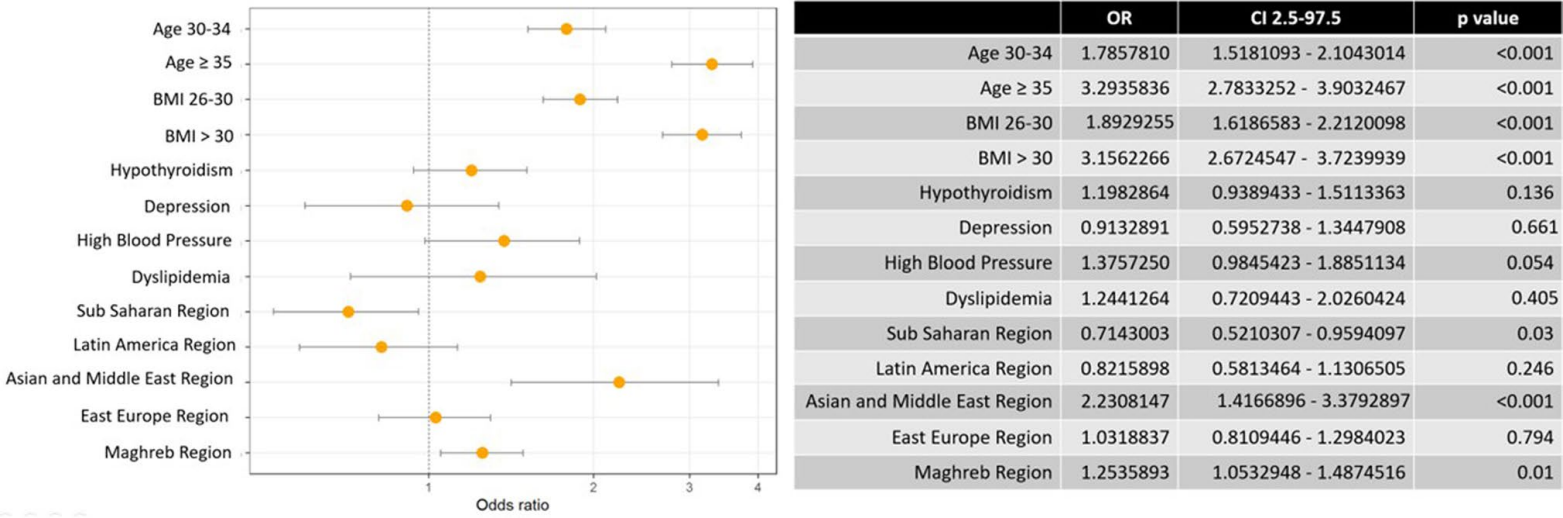
Multivariate analysis of the association of maternal risk factors with gestational diabetes

Finally, in Fig. 2, a multivariate analysis showed statistically significant differences, with a higher risk of gestational diabetes linked to: age of 30 years or older; BMI greater than or equal to 25; and origin from the Maghreb or Asia and the Middle East. Being of sub-Saharan origin has a protective factor for developing the GD. Other variables, such as hypothyroidism, high blood pressure, dyslipidaemia, and depression, were not significantly different between diabetics and non-diabetics.

## Discussion

In our sample of 17,177 pregnant women, we observed a prevalence of pre-gestational diabetes and GD of 8.2% and 6.5%, respectively. We found a relationship of gestational diabetes with different factors: age, with 6.8% in 30–34 year-old women and 11.3% in women over 35 (OR 1.78 and 3.29, respectively); overweight, with 8.29% (OR 1.89); and obesity, with 12.9% (OR 3.15). Finally, women from Asia and the Middle East and the Maghreb had a higher risk of diabetes, with 12.2% (OR 2.1) and 9.91% (OR 1.3), respectively, and Sub-Saharan women had a lower risk of it 6.07% (OR 0.71).

The latest studies show values of 8–25% of pregnancies with GD, with oscillations depending on the diagnostic criteria of the region and the population [32–34]. In our study, the prevalence was lower than in these ones (6.6%) and similar to others, such as the one conducted in the US with more than 12 million cases. Here, the age-adjusted GD ratio increased significantly from 47.6 to 63.5 per 1000 live births from 2011 to 2019.

Our multivariate analysis showed age and BMI as factors associated with diabetes, consistent with the study by Li et al. [11]. Moreover, Ferreira et al. identified gestational age and obesity as the two most important independent factors contributing to GD [35–38]. These along with family history of diabetes and multiple pregnancies are considered well-catalogued risk factors for GD. Finally, an Australian study estimated that 8.6%, 15.6%, and 19.5% of GDs could have been prevented by eliminating overweight, obesity, and maternal morbid obesity, respectively [39].

In our study, we found that 8.86% of diabetic pregnant women have hypertension, in comparison to 4.72% of non-diabetic ones, being these differences non-significative. Against with our results, Daly et al. found that women diagnosed with GD are twice as likely to develop hypertension [46].

With regard to dyslipidaemia, we did not observe any association with GD. On the contrary, Baumfeld et al. [47] found an increased risk of preeclampsia and/or GD in women with low HDL cholesterol and high pre-conception levels of triglycerides. O’Malley et al. concluded that the epidemiological association between GD and dyslipidaemia is mediated by maternal obesity, as women exclusively with obesity or GD did not have a high risk of dyslipidaemia in comparison to the rest [48]. A recent systematic review with meta-analysis considered the elevation of blood triglycerides to be the most important risk factor for GD. Similarly, high levels of total cholesterol, LDL, and VLDL; high triglycerides/HDL ratio; and low levels of HDL were found to be more common in the GD group [49]. In addition, the study by Diboun et al. provides data on metabolic impairment in women during the second trimester, identifying significant associations between the number of different metabolites (glutamate, branched amino acids, phosphatidylcholines and some triglycerides) [50] and diabetes mellitus 2 and GD.

Different articles associated depression with diabetes. Numerous studies either examined if diabetes was a risk factor for developing depression or if depression was a risk factor for diabetes during pregnancy; however, there is no clear consensus [51]. In particular, in our study, we did not observe this association. However, a systematic review and meta-analysis of recent cohort studies [52] concluded that women with pre-gestational and gestational diabetes had a statistically significant risk of prepartum depression, in comparison to women without diabetes.

Finally, regarding the country of origin, we observed that 14.4% of Asian pregnant women developed diabetes. This value coincides with the one found in other studies, such as the one conducted by Hod et al., in which they observed that 13% of women in China developed GD [53]. Another meta-analysis showed that the prevalence of GD in Asia was 20.9% [27–29]. In general, the prevalence of GD is increasing globally, with different studies predicting that 90% of new GD cases will occur in Asia [22–24]. An increase in the prevalence of GD among Asian and Moroccan women is indeed shown in several other studies, such as the one by Shah et al. [54, 55]. They studied the non-Hispanic Asian population/Pacific islands in the US (which included India, China, the Philippines, Japan, Korea, and Vietnam), and observed that the GD ratio significantly increased from 69.9 to 102.7 per 1000 live births per year from 2011 to 2019 [56]. There are some studies that demonstrate a higher risk of GD in Sub-Saharan woman; however, in our study, we observed a lower risk to develop it [57].

Our study has some limitations. First, the analysis was extracted from a database for clinical and administrative purposes. However, the National Health System is public, so the entire population has free access to Primary Care, Obstetrics services, and complementary tests, and all pregnant women can be screened. Second, our database does not include covariates such as history of diabetes in the family, diabetes history, history of previous impaired fasting plasma glucose, impaired glucose tolerance, macrosomia, thyroid autoimmunity and socioeconomic status and lifestyle (diet and physical activity), which are also risk factors for diabetes. Nevertheless, our multivariate analysis included all types of diabetes during pregnancy, involving heterogeneous subgroups.

## Conclusions

In this study, we found an association of diabetes during pregnancy with age and obesity, and a higher prevalence in the immigrant population from Asia and the Maghreb. The origin from Sub-Saharan Region had a protective effect to develop GD.

The high prevalence of diabetes during pregnancy poses a major challenge to prevent sequelae for both the mother and the baby, so it should be considered a public health priority. It is essential to prioritise preventive health care for women both before conception and during pregnancy by reducing risk factors.

We consider that more studies are needed to provide information on diabetes control, associated risk factors, and most susceptible populations, to finally provide better medical, family, and social intervention.

## Data Availability

Unidentified survey data will be provided by sending an email request to miriam_oros@hotmail.com.
